# Evaluating the necessity of colonoscopy in patients under 40 with rectal bleeding: insights from a large-scale retrospective analysis

**DOI:** 10.1007/s00384-024-04784-8

**Published:** 2024-12-16

**Authors:** Ibrahim M. Obeidat, Yousef Yahia, Prem Chandra, Amani Altaiam, Ethar Mohamed, Husam Saffo, Raya Abualsuod, Ala’a Al-deen Mousa, Duha Shalatouni, Khaled Alsa’ed, Mahmoud Y. Arabyat

**Affiliations:** 1 Gastroenterology and Hepatology Department, Hamad Medical Corporation, Doha, Qatar; 2 Medical Research Centre, Hamad Medical Corporation, Doha, Qatar; 3 Family Medicine Department, Primary Health Care Corporation, Doha, Qatar; 4 Internal Medicine Department, Hamad Medical Corporation, Doha, Qatar

**Keywords:** Bleeding per rectum, Colonoscopy, Young adults, Advanced adenoma, Colorectal cancer, Screening guidelines

## Abstract

**Purpose:**

Bleeding per rectum (BPR) is a common clinical presentation, and colonoscopy is the gold standard for evaluating patients aged ≥ 45 years. However, its role in younger patients remains unclear. This study evaluated the appropriateness of colonoscopy in patients < 40 years of age who presented with BPR.

**Methods:**

This retrospective observational study was conducted over 10 years, including 3422 patients aged 18–40 years who underwent colonoscopy for BPR. The cohort was divided into two age groups: younger (aged 18–30 years) and older (31–40 years). The patients’ baseline characteristics, colonoscopy findings, and histopathological results were analyzed.

**Results:**

Hemorrhoids were the most common finding (48%), with a higher prevalence in younger age groups (50.7%). Polyps were detected in 12.5% of patients, with 1.75% having advanced adenoma polyps (AAP) and 1.3% diagnosed with colorectal cancer (CRC). A family history of CRC/AAP was significantly associated with increased CRC risk (adjusted OR 6.35, 95% CI 2.24–18.02, *p* = 0.001) in explorative logistic regression analysis.

**Conclusion:**

AAP and CRC were detected in a small but significant proportion of patients, particularly among those aged 18–30 years. The detection of significant lesions in this age group highlights the need for targeted colonoscopy based on specific risk factors such as family history and clinical presentation. Future research should prioritize the creation of targeted assessment models to improve clinical decision making in this context.

**Supplementary Information:**

The online version contains supplementary material available at 10.1007/s00384-024-04784-8.

## Introduction

Bleeding per rectum (BPR) is a common clinical presentation encountered in the emergency department and outpatient settings. Studies have demonstrated that BPR affects a substantial proportion of the population, with prevalence rates ranging from 4 to 19% [1–3]. Colorectal cancer (CRC) is the third most prevalent cancer globally and the second leading cause of cancer-related mortality in 2020 [4]. Given the increasing incidence of colon cancer in younger individuals, as well as the higher incidence of neoplastic polyps in this demographic, a comprehensive assessment of this population is imperative [4–6]. While colonoscopy is the gold standard for evaluating patients aged ≥ 45 years, there is currently no consensus regarding the optimal approach for younger patients presenting with BPR [7]. Although most cases of BPR resolve on their own, it is essential to handle the diagnostic process with prudence, considering the need to identify colon lesions at an early stage while avoiding unnecessary invasive procedures. Colonoscopy provides a comprehensive view of the entire colon and terminal ileum, allowing for diagnosis and possible intervention of potential lesions. However, this procedure comes at the cost of an increased caseload and the need for complete colon preparation, which can lead to complications such as dehydration, electrolyte imbalance, and financial burden on the healthcare system [8, 9]. For patients aged < 40 years, the necessity and yield of colonoscopy remains a subject of debate, with few studies addressing this issue. A recent meta-analysis did not reach a definitive conclusion for this age group, highlighting the need for further studies with age stratification to examine the need for colonoscopy in patients aged < 40 years with BPR [10].

This retrospective descriptive study aimed to examine our institution’s experience in diagnosing and managing BPR in patients aged < 40 years old. This study will focus on patient characteristics, laboratory test results, colonoscopy findings, and histopathology results and will evaluate the effectiveness of colonoscopy in this population. The goal was to provide essential insights to guide clinical practice and to influence future screening recommendations. These insights are critical for healthcare providers managing BPR in younger patients as they strive to balance the diagnostic accuracy and appropriateness of the procedures.

## Method

### Study population and design

This retrospective study was conducted at our institution between January 2014 and January 2024. Our institution houses the leading endoscopy center in the nation and performs more than 14,000 endoscopic procedures annually. The institutional protocol requires that every patient presenting with rectal bleeding undergo at least one complete colonoscopy. Subsequent management was determined based on clinical and colonoscopic findings. All colonoscopies were performed by experienced endoscopists or supervised fellows using either the Pentax® or Fujifilm® high-definition colonoscopes. Informed consent was obtained from all the patients prior to the procedure. The inclusion criterion for this study was patients aged 18–40 years who had undergone colonoscopy for rectal bleeding. Patients were excluded from the study if they had known colon cancer, suspected Lynch syndrome or hereditary polyposis syndrome, inflammatory bowel disease, massive upper gastrointestinal bleeding, known case of chronic liver disease, or chronic kidney disease. Additionally, patients who did not achieve adequate bowel preparation, as indicated by a Boston bowel preparation scale score of 2 or 3 in each segment, were excluded from the study, unless the procedure was repeated with proper preparation. Medical records were reviewed to obtain demographic information and relevant risk factors such as smoking history and family history of colon cancer. The study population was stratified into two age cohorts: younger (aged 18–30 years) and older (31–40 years). Colonoscopy findings were documented with respect to the type, location, and size of the detected lesions whenever necessary. Procedure-related complications were recorded. An advanced adenoma polyp (AAP) was defined as meeting any of the following criteria: (I) tubular adenomas ≥ 1 cm; (II) adenomas with villous features or high-grade dysplasia, regardless of size; (III) sessile serrated polyps (SSP) ≥ 1 cm; (IV) traditional serrated adenomas of any size.

### Statistical considerations and data analysis

For continuous variables, data are presented as the mean and standard deviation (SD). Categorical variables were expressed as frequencies and percentages. The chi-squared test or Fisher’s exact test, as appropriate, was used to analyze categorical data and proportions. Continuous data were compared using the independent samples *t*-test for normally distributed data, whereas the Mann–Whitney *U* test was used for non-normally distributed data. Statistical significance was set at *p* < 0.05. Univariate and multivariate logistic regression methods were used to assess the predictive value of various potential predictors (age, sex, ethnicity, family history of CRC/AAP, smoking status, and hemoglobin (Hb) levels) associated with the outcome variables of CRC, AAP, and AAP/CRC (yes/no). For multivariate regression models, variables were considered if statistically significant at *p* < 0.10 level in univariate analysis or if determined to be clinically important.

All statistical analyses were conducted using the statistical packages SPSS version 29.0 (Armonk, NY, IBM Corp). All methods adhered to the Declaration of Helsinki and the hospital guidelines and regulations. The ethical committee waived the requirement for consent as only anonymous data without patient identifiers were provided to the research team. The study was approved by the Ethics and Research Committee of the Medical Research Center of Hamad Medical Corporation, Doha, Qatar (approval number MRC −01–24–232).

## Results

**Fig. 1:**
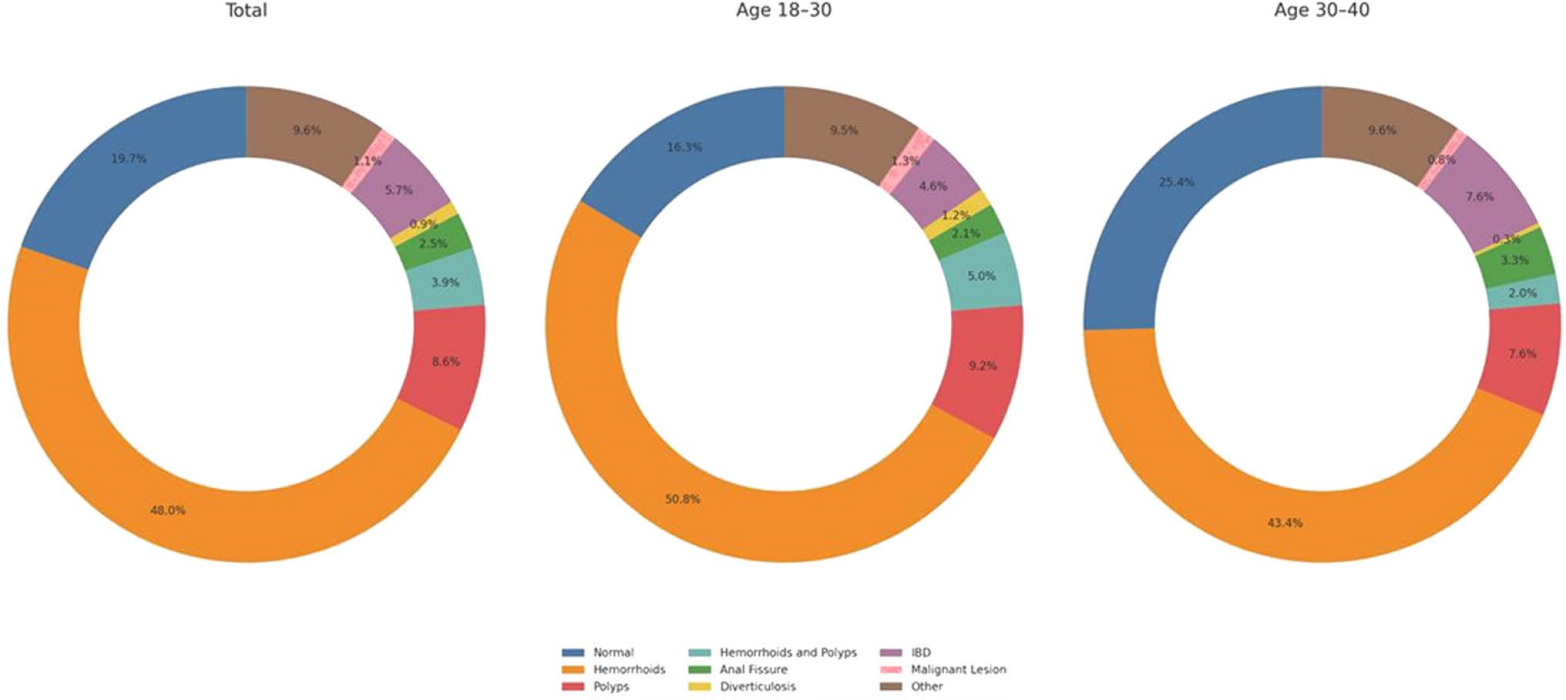
Distribution of colonoscopy findings in the total population, and in subgroups based on age (18–30 years and 30–40 years). The three charts illustrate the percentage of various colonoscopy findings, including normal findings, hemorrhoids, polyps, and other conditions, across these populations

Over a 10-year period, 3422 patients aged < 40 years who presented with rectal bleeding underwent colonoscopy. The patient cohort was stratified into two age groups: a younger group aged 18–30 years (*n* = 2153, 62.9%) and an older group aged 30–40 years (*n* = 1269, 37.1%). The baseline patient characteristics and blood test results are presented in Table 1 and Fig. 1. The cohort was predominantly male (70.5%), with males significantly more common in the younger group (73.2% vs. 65.9%, *p* < 0.001). The ethnic distribution varied; Middle Eastern patients were more prevalent in the older group (47% vs. 38.6%), whereas Southeast Asians were more common in the younger group (48.7% vs. 41.5%). In the cohort, 17.6% were smokers, with a significantly higher rate in the younger group (27.5% vs. 19.3%, *p* < 0.001). A family history of CRC was reported in 2.7% of the study population (5% in the younger age group vs. 3.5% in the older age group; *p* = 0.106). The mean hemoglobin level was almost similar in both age groups (13.05 g/dL ± 2.64 vs 13.07 g/dL ± 2.67, *p* = 0.822).

**Table 1:** Baseline characteristics of included patients

Characteristics	Total (*n* = 3422)	Age 18–30 years (*n* = 2153)	Age 30–40 years (*n* = 1269)	*p*-value
Gender: male, *n* (%)	2411 (70.5)	1575 (73.2)	836 (65.9)	< 0.001
Ethnicity, *n* (%)				< 0.001
Middle East	1427 (41.7)	831 (38.6)	596 (47)	
Southeast Asian	1574 (46)	1048 (48.7)	526 (41.5)	
Others	419 (12.2)	273 (12.7)	146 (11.5)	
Smoking, yes, *n* (%)	603 (17.6)	433 (27.5)	170 (19.3)	< 0.001
Family history of CRC/AAP, yes, *n* (%)	91 (2.7)	65 (5)	26 (3.5)	0.106
Hb (g/dL), mean ± SD	13.06 ± 2.66	13.05 ± 2.64	13.07 ± 2.67	0.822
Colonoscopy findings				
Normal	673 (19.7)	351 (16.3)	322 (25.4)	< 0.001
Hemorrhoids	1642 (48)	1091 (50.7)	551 (43.4)	< 0.001
Polyps	295 (8.6)	199 (9.2)	96 (7.6)	0.091
Hemorrhoids and polyps	133 (3.9)	108 (5)	25 (2)	0.001
Anal fissure	87 (2.5)	45 (2.1)	42 (3.3)	0.029
Diverticulosis	30 (0.9)	26 (1.2)	4 (0.3)	0.007
IBD	196 (5.7)	99 (4.6)	97 (7.6)	0.002
Malignant lesion	38 (1.1)	28 (1.3)	10 (0.8)	0.167
Other	328 (9.6)	206 (9.5)	122 (9.6)	0.965

Information on smoking status and family history of CRC/AAP were unavailable/missing (approx. 30–35% of the cases) in the patients’ record files, and thus the respective percentages values were computed using non-missing values

Colonoscopy revealed hemorrhoids as the sole finding in 48% of the cohort, with a higher prevalence in the younger group (50.7%) than in the older group (43.4%) (*p* < 0.001). Polyps were the only finding in 8.6% of the patients, while 3.9% of the study population had hemorrhoids in addition to polyps. Malignant lesions were detected in 1.1% of the patients and did not significantly differ between the age groups (*p* > 0.05). Other colonoscopic findings, such as solitary rectal ulcer syndrome or non-specific colitis, were reported in 9.6% of patients (Table 1). Notably, the rate of procedural complications, including post-polypectomy bleeding, perforation, and anesthesia-related side effects, was 0% in the entire cohort. It is important to note that because of the retrospective nature of the study and the fact that patient records date back up to 10 years, up to 30–35% of cases had missing values for smoking and a family history of CRC/AAP.

**Fig. 2:**
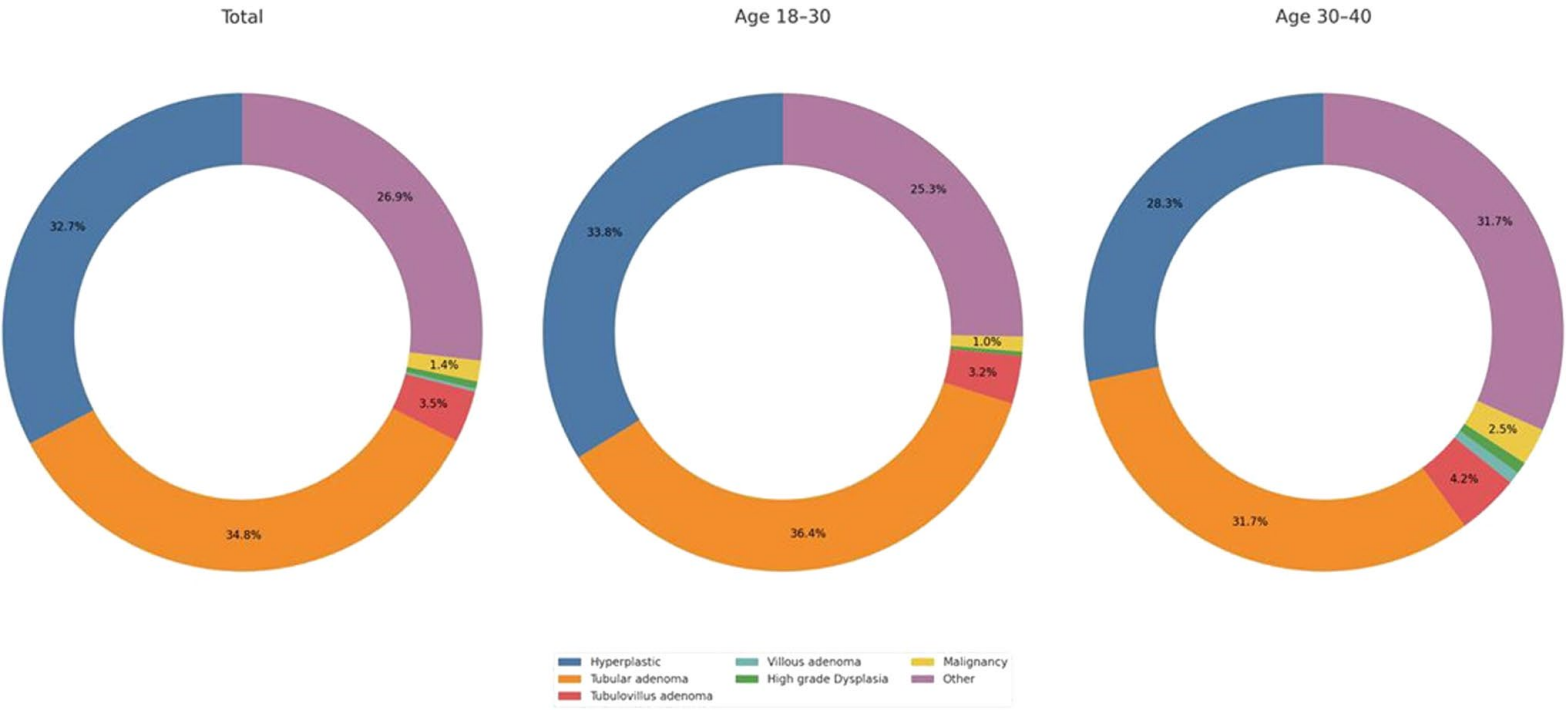
Distribution of polyp histology in the total population and in subgroups based on age (18–30 years and 30–40 years). The three pie charts illustrate the percentage of various polyp histology types, including hyperplastic polyps, tubular adenomas, tubulovillous adenomas, and others, across the different populations

**Fig. 3:**
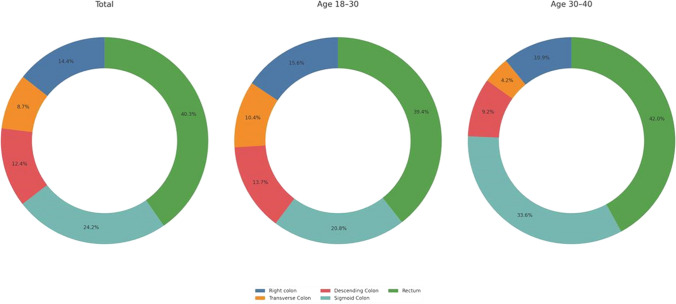
Distribution of polyp locations in the total population and in subgroups based on age (18–30 years and 30–40 years). The three pie charts illustrate the percentage distribution of polyps located in the right colon, transverse colon, descending colon, sigmoid colon, and rectum across the different populations

Regarding polyp characterization, diminutive polyps (1–5 mm) were the most prevalent in the cohort (62.8%) and were more frequently observed in the younger age group (67.8%) than in the older group (50.0%). Conversely, large polyps (≥ 10 mm) were more common in the older age group (26.7%) than in the younger age group (13.3%). Regarding polyp location, rectal polyps were the most prevalent (40%) and were similarly distributed across both groups (*p* > 0.05). Sigmoid colon polyps occurred more frequently in the older group (33.6% vs. 20.8%, *p* = 0.027). Other sites, including the right, transverse, and descending colon, accounted for 35.2% of cases. Histopathological examination of the polyps revealed that hyperplastic polyps (32.7%) and tubular adenomas (34.8%) were the most common and slightly more frequent in the younger group. The rate of malignant polyps was 1.4%, with no significant variation between the age groups. Further details of the polyp characteristics are presented in Table 2 and Figs. 2 and 3.

**Table 2:** Characteristics of polyps

Characteristics	Total (*n* = 428)	Age 18–30 years (*n* = 307)	Age 30–40 years (*n* = 121)	*p*-value
Polyp size				0.001
1–5 mm (diminutive)	269 (62.8)	209 (67.8)	60 (50.0)	
6–9 mm (small)	86 (20.09)	58 (18.8)	28 (23.3)	
≥ 10 mm (large)	73 (17.05)	41 (13.3)	32 (26.7)	
Polyp location				0.027
Right colon	61 (14.3)	48 (15.6)	13 (10.9)	
Transverse colon	37 (8.6)	32 (10.4)	5 (4.2)	
Descending colon	53 (12.3)	42 (13.7)	11 (9.2)	
Sigmoid colon	104 (24)	64 (20.8)	40 (33.6)	
Rectum	171 (40)	121 (39.4)	50 (42)	
Polyp histology				0.272
Hyperplastic	140 (32.7)	106 (33.4)	34 (28.3)	
Tubular adenoma	149 (34.8)	111 (36.0)	38 (31.7)	
Tubulovillous adenoma	15 (3.5)	10 (3.2)	5 (4.2)	
Villous adenoma	1 (0.2)	0 (0.0)	1 (0.8)	
High-grade dysplasia	2 (0.5)	1 (0.3)	1 (0.8)	
Malignancy	6 (1.4)	3 (1.0)	3 (2.5)	
Other	115 (26.9)	77 (25.0)	38 (31.7)

Given the potential risk of malignant transformation in AAP, a subgroup analysis of this population was conducted (Table 3). The study found that 60 patients (14%) in the cohort with polyps had AAP, males accounted for 75% of this subgroup, and smoking was reported in 32.6%, though more commonly in the younger group (42.3% vs. 17.6%, *p* = 0.092). Family history was reported in 7.9% of the patients. AAPs were predominantly located distal to the sigmoid colon (70%), but the proximal location was slightly more common in the younger group (22.3% vs. 4.2%, *p* = 0.056). A subsequent subgroup analysis of the 45 patients diagnosed with CRC was performed (Table 4). Consistent with the AAP subgroup, the majority of these patients were male, with a smoking status and family history of AAP/CRC reported in 11.1% and 11.1% of patients, respectively, with no statistically significant difference between the younger and older age groups (*p* > 0.05). The tumor was located proximal to the sigmoid colon in 13.3% of cases.

**Table 3:** Baseline characteristics of patients with advanced adenoma polyps

Characteristics	Total (*n* = 60)	Age 18–30 years (*n* = 36)	Age 30–40 years (*n* = 24)	*p*-value
Gender: male, *n* (%)	45 (75.0)	28 (77.8)	17 (70.8)	0.543
Ethnicity, *n* (%)				0.914
Middle East	26 (43.3)	15 (41.7)	11 (45.8)	
Southeast Asian	27 (45)	17 (47.2)	10 (41.7)	
Others	7 (11.7)	4 (11.1)	3 (12.5)	
Smoking, yes, *n* (%)	14 (32.6)	11 (42.3)	3 (17.6)	0.092
Family history of CRC/AAP, yes, *n* (%)	3 (7.9)	3 (15.8)	0 (0.0)	0.230
Location, proximal to sigmoid colon, *n*(%)	9 (30)	8 (22.3)	1 (4.2)	0.056
Hb (g/dL), mean ± SD	13.5 ± 2.63	13.75 ± 2.74	13.1 ± 2.42	0.354

Information on smoking status and family history of CRC/AAP were unavailable/missing (approx. 30–35% of the cases) in the patients’ record files, and thus the respective percentages values were computed using non-missing values

**Table 4:** Baseline characteristics of patients with colorectal cancer

Characteristics	Total (*n* = 45)	Age 18–30 years (*n* = 32)	Age 30–40 years (*n* = 13)	*p*-value
Gender: male, *n* (%)	32 (71)	23 (71.9)	9 (69.2)	0.859
Ethnicity, *n* (%)				0.778
Middle East	13 (28.9)	9 (28.1)	4 (30.8)	
Southeast Asian	26 (57.8)	18 (56.3)	8 (61.5)	
Others	6 (13.3)	5 (15.6)	1 (7.7)	
Smoking, yes, *n* (%)	5 (11.1)	4 (16)	1 (10)	0.647
Family history of CRC/AAP, yes, *n* (%)	5 (11.1)	4 (18.2)	1 (11.1)	0.627
Location, proximal to sigmoid colon, *n*(%)	6 (13.3)	5 (15.7)	1 (7.7)	0.478
Hb (g/dL), mean ± SD	12.31 ± 2.19	12.22 ± 2.29	12.55 ± 1.20	0.651

Information on smoking status and family history of CRC/AAP were unavailable/missing (approx. 30–35% of the cases) in the patients’ record files, and thus the respective percentages values were computed using non-missing values

An exploratory statistical analysis using logistic regression was conducted to evaluate and identify potential predictors and risk factors associated with AAP. Predictors in the younger age group, family history of CRC/AAP, and smoking status (yes) were observed to have an increased risk (adjusted OR ranged between 1.4 and 1.9) associated with AAP; however, their differences were statistically insignificant (*p* > 0.05). Univariate logistic regression analysis performed on the outcome variable of CRC revealed that a family history of CRC/AAP (yes) was associated with a significantly increased risk of CRC (unadjusted OR 4.50, 95% CI 1.68–12.07, *p* = 0.003). This association was confirmed in multivariate analysis (adjusted OR 6.35, 95% CI: 2.24–18.02, *p* = 0.001), adjusting for other potential predictors stated above. Other variables, including age, sex, and ethnicity, were not significantly associated with CRC in either univariate or multivariate logistic regression analyses. Further details are provided in Table 5 and Table 6.

**Table 5:** Logistic regression analysis: potential predictors for advanced adenoma polyps (AAP)

Parameter	Univariate OR (95% CI)	Univariate *p*-value	Multivariate OR (95% CI)	Multivariate *p*-value
Age 18–30 years	1.0 (0.63–1.80)	0.80	1.75 (0.87–3.53)	0.15
Gender (female)	0.7 (0.42–1.39)	0.39	1.0 (0.41–2.40)	0.99
Middle Eastern	0.96 (0.55–1.65)	0.88	1.12 (0.51–2.44)	0.76
Southeast Asian	0.95 (0.40–2.20)	0.90	1.4 (0.52–4.16)	0.46
Family history (yes)	2.0 (0.60–6.68)	0.25	1.88 (0.42–8.24)	0.40
Smoking (yes)	1.5 (0.80–2.91)	0.19	1.4 (0.65–3.29)	0.35
Hb level (g/dL)	1.0 (0.96–1.20)	0.18	1.1 (0.93–1.29)	0.23

Area under the curve (AUC) = 0.64 (95% CI 0.56, 0.72) computed using receiver-operating characteristics curve (ROC) analysis

**Table 6:** Logistic regression analysis: potential predictors for colorectal cancer (CRC)

Parameter	Univariate OR (95% CI)	Univariate *p*-value	Multivariate OR (95% CI)	Multivariate *p*-value
Age 18–30 years	0.65 (0.34–1.25)	0.19	0.91 (0.40–2.05)	0.82
Gender (female)	0.94 (0.49–1.80)	0.86	0.89 (0.39–2.04)	0.78
Middle Eastern	1.85 (0.95–3.62)	0.07	1.83(0.76–4.40)	0.17
Southeast Asian	1.63 (0.61–4.31)	0.33	1.65 (0.49–5.51)	0.41
Family history (yes)	4.50 (1.68–12.07)	0.003	6.35 (2.24–18.02)	0.001
Smoking (yes)	0.53 (0.20–1.37)	0.19	0.47 (0.13–1.65)	0.24
Hb level (g/dL)	0.92 (0.83–1.01)	0.08	0.91 (0.79–10.34)	0.14

Area under the curve (AUC) = 0.72 (95% CI 0.62, 0.81) computed using receiver-operating characteristics curve (ROC) analysis

Additionally, an exploratory statistical analysis using multinomial logistic regression was performed to evaluate and assess potential predictors of risk factors associated with each colonoscopy finding, considering normal colonoscopy findings as a reference group. These findings are presented in Supplementary Table 1.

## Discussion

This retrospective study assessed the need for colonoscopy in young patients who presented with BPR over a 10-year period. Hemorrhoids were identified as the most prevalent etiology of BPR in the cohort, consistent with the established understanding that hemorrhoidal bleeding is a primary cause of BPR [11–13]. CRC was identified in 1.3% of patients, while AAP were present in 1.75%. In contrast, Wong et al. reported a CRC prevalence of 0.4% and an AAP prevalence of 4.5% in their systematic review and meta-analysis [14]. The observed discrepancies may be attributed to variations in ethnicity and age. Notably, 46% of our study population consisted of individuals of Southeast Asian descent, and previous studies have indicated that the mean age at CRC onset in this group is lower than that in other populations [15, 16].

A significant proportion (86.7%) of CRC cases in our study was detected within the reach of sigmoidoscopy, a finding comparable to that of Khalid et al., in which 100% of CRC cases were accessible by sigmoidoscopy [13]. However, 39% of adenomas and 30% of AAP were proximal to the sigmoid colon. This underscores the necessity of performing complete colonoscopy rather than relying solely on sigmoidoscopy for comprehensive evaluation, a recommendation supported by numerous other studies [12, 17, 18].

We observed that the prevalence of AAP and CRC was notably higher in the younger age group than in the other groups, with rates of 60% and 71%, respectively. Although the baseline parameters were largely comparable between the two groups, this discrepancy may be attributed to unmeasured variables, such as genetic predisposition, which could contribute to accelerated colorectal carcinogenesis [19]. These findings emphasize the importance of thorough risk stratification and careful consideration of this age group when determining the appropriateness of colonoscopy. Although regression analysis was conducted as an exploratory analysis, the significant association between a family history of CRC/AAP and CRC (OR 6.35) warrants further attention. This finding reinforces the critical role of family history as a key factor in assessing younger patients with BPR. This finding is consistent with the established understanding that a positive family history significantly increases the risk of CRC even in individuals below the typical screening age [7].

This study has several strengths. First, the large sample size in this particular age group, compared to previous studies, allowed for a more comprehensive evaluation of young patients with rectal bleeding who underwent colonoscopy. Additionally, the diversity of our patient population, particularly in terms of ethnicity, enhances the generalizability of our findings. The strict inclusion criteria and the use of the procedure by experienced endoscopists further strengthened the reliability of the results. Finally, the stratification of patients by age cohort, along with a detailed analysis of polyp characteristics and histopathology, provides valuable insights into the effectiveness of colonoscopy in this population. However, our study has certain limitations. Primarily, its retrospective design may introduce biases, such as observer bias and unmeasured confounding factors. Additionally, reliance on medical records results in incomplete data on smoking and family history, which may limit the generalizability of the risk associations. Although the study was conducted at a single center by only gastroenterologists, this could potentially minimize the probability of performance bias.

The low prevalence of malignant lesions (1.3%) in patients under 40 years of age, although reassuring, should not diminish the importance of early onset CRC, which, though rare, has significant clinical consequences. The detection of AAP and CRC in this population highlights the necessity for colonoscopic evaluation, especially in individuals with a family history of CRC or other high-risk factors. With advancements in artificial intelligence, deep learning techniques, and progress in pathomics and genomics, future controlled trials on risk modeling in this age group could offer critical insights for optimizing clinical decision making. This would facilitate a careful balance between minimizing unnecessary procedures and ensuring that clinically significant lesions are not overlooked.

## Conclusion

In this retrospective study of young patients presenting with BPR, hemorrhoids were identified as the most common etiology. However, AAP and CRC were detected in a small but significant proportion of patients, particularly among those aged 18–30 years. The detection of significant lesions in this age group highlights the need for targeted colonoscopy based on specific risk factors, such as family history and clinical presentation, rather than the routine screening of all young patients with BPR. Early diagnostic intervention is essential for patients at high risk of misdiagnosis of malignant conditions. Future studies should focus on developing refined risk stratification models and personalized assessment tools to balance early detection with the risks and costs of unnecessary procedures, optimizing clinical decision making, and patient outcomes in this demographic group.

## Supplementary Information

Below is the link to the electronic supplementary material.

Supplementary Material 1 (DOCX 162 bytes)

## Data Availability

No datasets were generated or analysed during the current study.

## Abbreviations

BPR: Bleeding Per Rectum
AAP: Advanced Adenoma Polyp
CRC: Colorectal Cancer
Hb: Hemoglobin
SSP: Sessile serrated polyps
