# Supplementary material for: Evaluating the necessity of colonoscopy in patients under 40 with rectal bleeding: insights from a large-scale retrospective analysis
Source: Int J Colorectal Dis. 2024 Dec 16;39(1):202. doi: 10.1007/s00384-024-04784-8 (PMC11649774; doi:10.1007/s00384-024-04784-8)
Supplement: Supplementary file 1 — Supplementary Material 1 (DOCX 162 bytes) [file 384_2024_4784_MOESM1_ESM.docx]

| **Colonoscopy Findings** | **Predictors** | **Adjusted Odds ratio (OR)** | **95% Confidence Interval**  **for odds ratio (OR)** | | **P-value** |
| --- | --- | --- | --- | --- | --- |
|  |  |  | **Lower Bound** | **Upper Bound** |  |
| **Internal/External Hemorrhoids** |  |  |  |  |  |
|  | Age: 31-40 years^*^ | 1.67 | 1.30 | 2.16 | **<0.0001** |
|  | Age: 18-30 years | reference |  |  |  |
|  | Gender: Male | 1.27 | 0.97 | 1.67 | 0.084 |
|  | Gender: Female | reference |  |  |  |
|  | Nationality: Middle eastern | 0.55 | 0.36 | 0.85 | **0.007** |
|  | Nationality: Asian | 0.78 | 0.50 | 1.20 | 0.256 |
|  | Nationality: Others | reference |  |  |  |
|  | Smoking: Yes | 1.21 | 0.87 | 1.68 | 0.256 |
|  | Smoking: No | reference |  |  |  |
|  | Family Hx of CRC/AAP: Yes | 2.07 | 0.95 | 4.52 | **0.068** |
|  | Family Hx of CRC/AAP: No | reference |  |  |  |
| **Polyps** |  |  |  |  |  |
|  | Age: 31-40 years^δ^ | 1.30 | 0.88 | 1.93 | 0.191 |
|  | Age: 18-30 years | reference |  |  |  |
|  | Gender: Male | 1.24 | 0.81 | 1.91 | 0.326 |
|  | Gender: Female | reference |  |  |  |
|  | Nationality: Middle eastern | 0.68 | 0.35 | 1.32 | 0.256 |
|  | Nationality: Asian | 1.03 | 0.53 | 2.00 | 0.942 |
|  | Nationality: Others | reference |  |  |  |
|  | Smoking: Yes | 1.32 | 0.81 | 2.15 | 0.264 |
|  | Smoking: No | reference |  |  |  |
|  | Family Hx of CRC/AAP: Yes | 3.45 | 1.32 | 9.01 | **0.011** |
|  | Family Hx of CRC/AAP: No | reference |  |  |  |

**Supplementary Table 1:** Multinomial logistic regression analysis to evaluate and assess predictive value of potential predictors for each respective colonoscopy finding considering normal colonoscopy finding as a reference group.

**Supplementary Table 1. Contd….**

| **Hemorrhoids and Polyps** |  |  |  |  |  |
| --- | --- | --- | --- | --- | --- |
|  | Age: 31-40 years^*^ | 2.75 | 1.58 | 4.77 | **0.001** |
|  | Age: 18-30 years | reference |  |  |  |
|  | Gender: Male | 1.80 | 1.02 | 3.20 | **0.044** |
|  | Gender: Female | reference |  |  |  |
|  | Nationality: Middle eastern | 0.66 | 0.30 | 1.47 | 0.306 |
|  | Nationality: Asian | 0.93 | 0.42 | 2.06 | 0.859 |
|  | Nationality: Others | reference |  |  |  |
|  | Smoking: Yes | 1.76 | 1.02 | 3.03 | **0.043** |
|  | Smoking: No | reference |  |  |  |
|  | Family Hx of CRC/AAP: Yes | 0.55 | 0.07 | 4.52 | 0.580 |
|  | Family Hx of CRC/AAP: No | reference |  |  |  |
| **Anal Fissure** |  |  |  |  |  |
|  | Age: 31-40 years | 0.56 | 0.31 | 1.02 | **0.059** |
|  | Age: 18-30 years | reference |  |  |  |
|  | Gender: Male | 0.95 | 0.49 | 1.81 | 0.865 |
|  | Gender: Female | reference |  |  |  |
|  | Nationality: Middle eastern | 0.81 | 0.29 | 2.28 | 0.691 |
|  | Nationality: Asian | 0.93 | 0.32 | 2.68 | 0.894 |
|  | Nationality: Others | reference |  |  |  |
|  | Smoking: Yes | 1.17 | 0.53 | 2.59 | 0.692 |
|  | Smoking: No | reference |  |  |  |
|  | Family Hx of CRC/AAP: Yes | na | na | na | 0.998 |
|  | Family Hx of CRC/AAP: No | reference |  |  |  |
| **Diverticulosis** |  |  |  |  |  |
|  | Age: 31-40 years^*^ | 5.28 | 1.53 | 18.23 | **0.008** |
|  | Age: 18-30 years | reference |  |  |  |
|  | Gender: Male | 1.01 | 0.39 | 2.64 | 0.985 |
|  | Gender: Female | reference |  |  |  |

**Supplementary Table 1. Contd….**

|  | Nationality: Middle eastern | 0.46 | 0.15 | 1.39 | 0.166 |
| --- | --- | --- | --- | --- | --- |
|  | Nationality: Asian | 0.23 | 0.06 | 0.85 | **0.027** |
|  | Nationality: Others | reference |  |  |  |
|  | Smoking: Yes | 1.96 | 0.73 | 5.22 | 0.179 |
|  | Smoking: No | reference |  |  |  |
|  | Family Hx of CRC/AAP: Yes | na | na | na |  |
|  | Family Hx of CRC/AAP: No | reference |  |  |  |
| **IBD** |  |  |  |  |  |
|  | Age: 31-40 years | 0.75 | 0.49 | 1.16 | 0.199 |
|  | Age: 18-30 years | reference |  |  |  |
|  | Gender: Male | 1.71 | 1.02 | 2.86 | **0.040** |
|  | Gender: Female | reference |  |  |  |
|  | Nationality: Middle eastern | 0.81 | 0.37 | 1.78 | 0.604 |
|  | Nationality: Asian | 1.11 | 0.51 | 2.42 | 0.799 |
|  | Nationality: Others | reference |  |  |  |
|  | Smoking: Yes | 1.49 | 0.88 | 2.53 | 0.138 |
|  | Smoking: No | reference |  |  |  |
|  | Family Hx of CRC/AAP: Yes | 0.52 | 0.06 | 4.19 | 0.535 |
|  | Family Hx of CRC/AAP: No | reference |  |  |  |
| **Cancer** |  |  |  |  |  |
|  | Age: 31-40 years^δ^ | 1.92 | 0.82 | 4.51 | 0.132 |
|  | Age: 18-30 years | reference |  |  |  |
|  | Gender: Male | 1.01 | 0.44 | 2.29 | 0.996 |
|  | Gender: Female | reference |  |  |  |
|  | Nationality: Middle eastern | 0.62 | 0.16 | 2.37 | 0.486 |
|  | Nationality: Asian | 1.22 | 0.33 | 4.52 | 0.768 |
|  | Nationality: Others | reference |  |  |  |
|  | Smoking: Yes | 0.75 | 0.24 | 2.35 | 0.623 |
|  | Smoking: No | reference |  |  |  |
|  | Family Hx of CRC/AAP: Yes | 9.61 | 2.84 | 32.53 | **0.001** |
|  | Family Hx of CRC/AAP: No | reference |  |  |  |
|  |  |  |  |  |  |

**Supplementary Table 1. Contd….**

| **Other** |  |  |  |  |  |
| --- | --- | --- | --- | --- | --- |
|  | Age: 31-40 years | 1.09 | 0.75 | 1.58 | 0.639 |
|  | Age: 18-30 years | reference |  |  |  |
|  | Gender: Male | 1.49 | 0.98 | 2.26 | **0.059** |
|  | Gender: Female | reference |  |  |  |
|  | Nationality: Middle eastern | 0.68 | 0.37 | 1.25 | 0.214 |
|  | Nationality: Asian | 0.74 | 0.40 | 1.39 | 0.353 |
|  | Nationality: Others | reference |  |  |  |
|  | Smoking: Yes | 1.32 | 0.83 | 2.09 | 0.239 |
|  | Smoking: No | reference |  |  |  |
|  | Family Hx of CRC: Yes | 2.99 | 1.15 | 7.79 | **0.025** |
|  | Family Hx of CRC: No | reference |  |  |  |
| *Each colonoscopy finding was compared using reference group normal colonoscopy finding*  *na:not available/estimated* | | |  |  |  |

*It was observed that the older age group was significantly associated with higher risk of hemorrhoids (adjusted OR 1.67; 95% CI 1.30, 2.16; p<0.001), hemorrhoids and polyps (adjusted OR 2.75; 95% CI 1.58, 4.77; p=0.001) diverticulosis (adjusted OR 5.28; 95% CI 1.53, 18.23, p=0.008).

δSimilar trend in results were observed when compared age groups with other colonoscopy findings such as polyps and CRC, however the differences didn’t reach statistical significance (p>0.05).

Gender male was associated with higher risk in most of these colonoscopy findings (adjusted OR ranged between 1.24 to 1.80).

Family history of CRC/AAP (yes) was associated with significantly higher risk of polyps (adjusted OR 3.45; 95% CI 1.32, 9.01; p=0.011), CRC (adjusted OR 9.61; 95% CI 2.84, 35.23, p=0.001) and other colonoscopy findings (adjusted OR 2.99; 95% CI 1.15, 7.79, p=0.025).

Similar trend in results were observed when compared smoking status (yes) with each colonoscopy findings, however the differences didn’t reach statistical significance (p>0.05).
